# Intrapreneurial Self-Capital Mediates the Connectedness to Nature Effect on Well-Being at Work

**DOI:** 10.3390/ijerph16224359

**Published:** 2019-11-08

**Authors:** Annamaria Di Fabio, Letizia Palazzeschi, Mirko Duradoni

**Affiliations:** 1 Department of Education, Languages, Intercultures, Literatures and Psychology (Psychology Section), University of Florence, 50135 Firenze, Italy; letizia.palazzeschi@unifi.it; 2 Department of Information Engineering, University of Florence, 50139 Firenze, Italy; mirko.duradoni@unifi.it

**Keywords:** connectedness to nature, intrapreneurial self-capital (ISC), well-being at work, primary prevention perspective, promoting health among workers

## Abstract

Researchers are being called upon to find and explore viable solutions to protect the environment and promote health in the new digital era of the 21st century, since the rapid changes transpiring within our technological societies may be detrimental to workers but also offer opportunities for growth. The concept of connectedness to nature, on the one hand, is a proxy for important environmentally protective and responsible behaviors; on the other, it has been studied in relation to people’s well-being. To promote health, it is crucial to act from a primary prevention perspective, which is focused on finding variables that can be increased through specific training. In this framework, intrapreneurial self-capital (ISC) appears to be related both to people’s connectedness to nature and their well-being. This study analyzes exploratively the relationship between connectedness to nature, ISC, and well-being at work, since these variables have never been studied together. A mediation model is tested to assess whether ISC could mediate the relationship between connectedness to nature and workers’ well-being. The mediation analysis highlights that ISC, as a core of preventive resources, potentially sustains the effect of feeling connected to nature on well-being at work. Thus, interventions aimed at increasing and acquiring preventive resources, such as ISC, could be beneficial in protecting the environment and in promoting health among workers.

## 1. Introduction

The protection of the environment and the promotion of people’s well-being are not separate issues. The rapid and continuous changes that characterize our contemporary technological societies put both at risk [1,2]. Nevertheless, in line with the calls of the psychology of sustainability and sustainable development [3,4,5], the positive primary preventive perspective [2,6,7], and the United Nations (UN) [8], this new digital era also offers contexts and conditions for a paradigm shift to take place, focused on processes of personal growth and flexible change [1,2]. The importance of the psychological aspects that are related to sustainable development have also grown in importance in recent years [3]. For this reason, scholars are being called upon to find viable solutions that, on the one hand, can promote people’s well-being and adaptation to our everchanging societies, and on the other can manage and support sustainability processes and environmental protection [9,10]. To pursue these combined goals, the connectedness to nature construct [11,12] has recently been studied more deeply as not only a useful proxy for important environmentally favorable behaviors [12,13,14,15,16,17,18], but also as a way to reconcile people to the natural environment, thus benefiting both humans and ecosystems [19,20]. Connectedness to nature refers to how people identify both on cognitive and affective levels with the natural environment, and the relationships they form with nature [12]. Therefore, connectedness to nature can be defined on two main axes: the individuals’ cognitive representation of self [18] and their affective and experiential connection with nature [11].

Given the potential of connectedness to nature to address sustainability issues in a broader and more comprehensive manner, efforts have been made by scholars to identify psychological resources able to positively affect people’s connections and identification with the natural environment, in line with the primary prevention approach [2,6,7]. Indeed, primary prevention research is focused both on reducing risks and on building people’s strengths to adaptively cope with the demands of the current era. Among these psychological resources, intrapreneurial self-capital (ISC) appears to be particularly promising, since it entertains positive relationships with both well-being measures [21,22,23,24] and connectedness to nature [25]. ISC is a higher-order construct (i.e., a core of individual resources) that is defined by the following aspects: core self-evaluation, hardiness, creative self-efficacy, resilience, goal mastery, decisiveness, and vigilance [26]. People with a high level of ISC hold a positive self-evaluation of their own ability to identify with and commit themselves to significant objectives. Moreover, they usually feel in control over life events and can solve problems in a creative manner. They also succeed in changing constraints into resources, developing their own skills, and applying accurate and adaptive decisions to every life situation [26,27]. The relationship between ISC and other constructs theoretically similar in the literature, such as psychological capital, was empirically examined [28]. The results showed that ISC presents aspects of specificity with respect to psychological capital. In fact, if on one side these two constructs present aspects of similarity (both refer to individual resources useful for career and life management, assume the responsibility for individuals’ own actions in committing to success, and include aspects of self-efficacy), on the other side a crucial differentiation is maintained because ISC comprises aspects relative to decisional processes and adaptive vigilance not encompassed in psychological capital. ISC is thus also characterized by taking self-determined and critical decisions in an adaptive and creative way, overcoming possible constraints. Instead, the specificity of psychological capital [29] with respect to ISC refers to a positive psychological state that permits people to have experiences in the present, prefiguring future pathways and outcomes with optimism, and interiorizing positive elements of present experiences as motivation to answer to contextual stimuli and carry out the various activities.

The association between ISC and connectedness to nature emerged in a previous study [30], underlining the contribution of ISC as mediator in the relationship between extraversion and connectedness to nature. Individuals who successfully included nature in their own lives reported higher levels of ISC [30]. Since it emerged recently that ISC, as a primary preventive resource, appears to be able to support the achievement by workers of a deeper sense of connecting with nature [25], in this research we explore whether ISC can also mediate the expected outcomes in terms of well-being at work related to connectedness to nature. To accomplish this, we first tested the relationship between connectedness to nature and the well-being of workers in terms of the work and meaning inventory (WAMI). The WAMI [31] permits us to consider the different ways of defining and assessing the meaning of work (MOW), proposing a multidimensional model of work as a subjectively meaningful experience consisting of experiencing positive meaning in work, sensing that work is a key avenue for making meaning, and perceiving one’s work to benefit some greater good. For this reason, it represents a more comprehensive construct with respect to that proposed by the MOW team, [32] which is based on a more traditional definition of MOW [33]. The WAMI encompasses aspects that are not included in the MOW definition. For instance, the desire to make a positive impact on the greater good. In this sense, the WAMI may capture individuals’ propensity to expand themselves towards objectives other than those strictly pursued within the workplace, driving them towards higher levels of connectedness to nature [11]. Although the relationship between well-being and connectedness to nature appears to have been consolidated [19,20], it has never been tested using more specific and suitable predictors for workers. Similarly, past ISC results strictly related to workers’ and students’ well-being [21,22,23,24], but a gap still exists in reference to ISC and measures completely designed to capture well-being within the work environment.

We formulate the following hypotheses based on the literature:

**H1:**  *Connectedness to nature is positively correlated with the WAMI*

**H2:**  *ISC is positively correlated with the WAMI*

**H3:**  *ISC is positively correlated with connectedness to nature*

**H4:**  *ISC mediates the relationship between connectedness to nature and the WAMI*

## 2. Materials and Methods

### 2.1. Participants

For this study, we recruited 203 (131 females) participants, who worked in different public and private organizations. Their average age was 44.02 (standard deviation = 10.98).

Given the exploratory nature of the present work, the authors chose a non-probability method based on the voluntary census to test the hypotheses. In these circumstances, studies based on voluntary participation can be extremely effective [34].

Relying on the descriptive statistics and correlation matrices shown in previous similar studies, we determined the necessary sample size to conduct our analyses. Assuming the same relationship between the variables that we want to test in our mediation models and a significance level of 0.05, we carried out the Monte Carlo Power Analysis for Indirect Effects [35]. The analysis showed that a sample size of 108 individuals would be enough to ensure a statistical power of 0.80. We involved 203 participants to give us a power of 0.95—at the lower limit of the confidence interval. Participants were recruited with approximately a 1:1 ratio to test our hypotheses across the private and public sectors.

### 2.2. Measures

#### 2.2.1. Connectedness to Nature Scale (CNS)—Italian Version

The Italian version [36] of the CNS [11] uses 14 items measured using a five-point Likert-type scale (ranging from 1 = Strongly disagree to 5 = Strongly agree). The scale returns the individuals’ trait levels of feeling emotionally and cognitively connected to the natural world. High scores on the CNS signify a deeper connection with nature. The Italian version of the scale shows an adequate dimensionality (χ^2^/df = 2.16, Tucker–Lewis index (TLI) or non-normed fit index (NNFI) = 0.92, comparative fit index (CFI) = 0.94, root mean square error of approximation (RMSEA) = 0.07). The reliability coefficients for the CNS are respectively 0.91 for the Italian version and 0.84 for the original version. Examples of items include: “I often feel a sense of oneness with the natural world around me”, “I think of the natural world as a community to which I belong”, and “I have a deep understanding of how my actions affect the natural world”.

#### 2.2.2. The WAMI—Italian Version

The Italian version [37] of the WAMI [31] consists of 10 items measured using a seven-point Likert-type scale (ranging from 1 = Absolutely untrue to 7 = Absolutely true). The original scale holds good dimensionality indices (χ^2^ = 64.19, TLI or NNFI = 0.95, CFI = 0.96, RMSEA = 0.09, standardized root mean square residual (SRMR) = 0.05). The Italian version holds still-satisfactory fit indices (χ^2^/df = 2.84, TLI = 0.91, CFI = 0.92, RMSEA = 0.07, SRMR = 0.06). The Cronbach’s alpha for the total score of the original version is 0.93 [31]. Examples of items include: “I have found a meaningful career”, “My work helps me make sense of the world around me”, and “I know my work makes a positive difference in the world”.

#### 2.2.3. Intrapreneurial Self-Capital Scale (ISCS)—Italian Version

The ISCS [26] consists of 28 items measured using a five-point Likert-type scale to assess the ISC construct, which is defined by the following aspects: self-evaluation, hardiness, creative self-efficacy, resilience, goal mastery, decisiveness and vigilance. The reported Cronbach’s alpha coefficient for the ISCS total score is 0.86 for the Italian version. The psychometric properties of the scale were reported by Di Fabio [26]. ISCS holds appropriate and adequate dimensionality indices (χ^2^/df = 1.43, TLI or NNFI = 0.90, CFI = 0.90, RMSEA = 0.05, SRMR = 0.04).

### 2.3. Procedure

The questionnaires were administered to the workers in group sessions by trained psychologists in line with Italian law’s requirements of privacy and informed consent (Law Decree DL-101/2018) and EU regulation (2016/699). If they wanted to, participants could withdraw from the data collection at any time. The questionnaires were administered in counterbalanced order to control for order effects.

### 2.4. Data Analysis

We verified the preconditions necessary for mediation analysis. Indeed, we assessed the variables’ normality, homoscedasticity, and linearity. Moreover, common-method bias was assessed with Harman’s Single-Factor Test [38]. According to the rules commonly used for the Pearson’s r interpretation (Hinkle, Wiersma, & Jurs, 2003) we have negligible correlation for coefficient ≤30, low correlation for values between 0.30 and 0.50, moderate correlation for r values ranging from 0.50 to 0.70, high correlation for coefficients between 0.70 and 0.90, and very high correlation for values ranging from 0.90 to 1.00. In our case, it is important to notice that the relationship between ISC and other well-being measures (for instance in Di Fabio & Duradoni’s review work, 2019) are moderate and never resulted higher than 0.70, which is considered the threshold for suspecting a consistent and biasing overlap between constructs. For all the variables suitable for the mediation analysis, we examined gender-related differences by means of the Student t test and Cohen’s d coefficient. Subsequently, regression procedures recommended by Hayes [39] were performed for the assessment of mediation using PROCESS version 3.2 [40]. Mediation processes investigate intermediate variables (i.e., mediators (M)) and their effects on the relationship between an independent variable (X) and an outcome variable (Y). For simple mediation models (i.e., theoretical model 4) three variables are needed (i.e., M, X, Y). According to Hayes [39], in a simple mediation model there are two possible pathways in which X affects Y. The first path directly links X with Y, while the second connects X and Y indirectly through M. Complete or full mediation occurs when variable X no longer significantly affects Y after M has been introduced in the model, while partial mediation happens when the direct path between X to Y is reduced in size but is still statistically significant. We proceeded to estimate the effect size of the mediator (i.e., how much of the independent variable’s effect was accounted for) and of the entire model (i.e., direct and indirect pathways effects).

## 3. Results

### 3.1. Descriptive Statistics

The descriptive statistics for our sample are presented in Table 1, which includes all the observables accounted for in our data collection.

### 3.2. Mediation Assumptions and Gender Differences

Before proceeding with mediation analysis, we explored the correlation between CNS, WAMI, and ISCS scores by means of Pearson’s r coefficient.

As shown in Table 2, ISCS correlates positively with both CNS and WAMI. Moreover, CNS shows a positive linear relationship with WAMI, which signals that workers who report higher levels of connectedness to nature also experience higher positive meaning in their work. All the correlations mentioned reach a medium correlation magnitude [41]. Overall, the three variables considered (i.e., CNS, WAMI and ISCS) appear to be suitable for mediation analysis. Gender-related effects on CNS, WAMI, and ISCS were investigated before proceeding with mediation analysis.

As we can gather from Table 3, gender does not appear to affect CNS ($t_{\left(201\right)}=\;0.46;p=0.64),$ WAMI ($t_{\left(201\right)}=\;-0.96;p\;=0.34)$ and ISCS ($t_{\left(201\right)}=\;1.75;p=0.08)$ significantly. Moreover, the gender-related effect size upon these variables is small [41]. Since gender does not seem to affect the three variables selected for mediation analysis in a statistically significant way, the researchers excluded gender from the subsequent mediation modeling analysis.

### 3.3. Mediation Analysis

**Figure 1:**
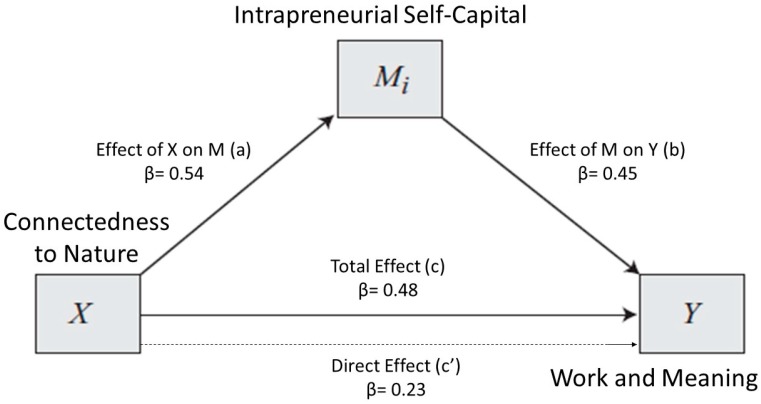
Model 1. Relationship between connectedness to nature and the WAMI, with ISC as a mediator. Mediator Effect Size = 0.11.

Given the previous results, we decided to proceed with the mediation analysis considering CNS as an independent variable and ISCS as a mediator to predict WAMI. The effect of CNS on the mediator (ISCS) is accounted for by path a, while path b statistics show how much the mediator affects the outcome variable (i.e., CNS). Path c’ and path c refer instead to the effect of the independent variable on the outcome variable when the mediator is respectively accounted for or not. Figure 1 shows the relationship between CNS and WAMI when ISCS is accounted for as a mediator. The statistics related to each mediation path are presented in Table 4.

In line with our predictions, CNS demonstrates a significant direct positive influence on WAMI (path c) and affects it indirectly through ISC. However, the direct effect (path c’) of CNS on WAMI does not appear to be statistically significant (i.e., when the mediator is accounted for). Workers with a high level of connectedness to nature also reported a higher level of ISC (path a), and participants who had high scores in ISC appeared to experience a higher positive meaning in their work (path b). In Table 5, model effects indices are summarized.

As we can gather from Table 5, the 11% of variance in the WAMI score is explained by the indirect effect (i.e., by using ISC as a mediator).

## 4. Discussion

In the present work, we present a mediation model that extends previous research by examining the relationship between people’s work-related predictor of well-being (i.e., the WAMI) and connectedness to nature, and by assessing the role of ISC as a mediator of such a relationship.

First, we confirm and expand the literature regarding connectedness to nature and well-being [19,20] by employing a designed measure able to capture an aspect of people’s well-being within the work environment. A positive correlation emerges between connectedness to nature and the WAMI as predicted by our first hypothesis (H1). The relationship between ISC and the WAMI is confirmed [21,22,23,24], showing that ISC is also positively related to the particular measure of well-being at work involved in our study (H2). Therefore, based on our results, the contribution of ISC to people’s well-being appears to be strengthened even more when work meaning is considered [42]. In line with the recent literature [25], our article highlights a positive linear relationship between ISC and connectedness to nature (H3). Finally, given the existence of such relationships, we carried out a mediation analysis. As predicted by hypothesis 4, ISC fully mediates the CNS effect on meaning at work (H4). Following the interpretation of the totally standardized indirect effect index [43], we obtain a medium mediator effect size for the proposed model. In line with Cohen’s guidelines [41], small, medium, and large effect sizes are defined respectively by 0.01, 0.09, and 0.25 values.

The fact that the direct effect in the mediation model (i.e., when the mediator is accounted for) becomes statistically insignificant highlights how ISC resources could be very promising in promoting the outcomes of connectedness to nature in terms of meaning at work.

In line with the self-expansion model [11,30], previous research suggests that ISC resources could ease the achievement of a deeper connectedness to nature [25]. Our results add a novel piece of information to this finding, highlighting that ISC resources are also potentially useful for supporting workers’ well-being at work due to the development of a new sense of connection and identification with nature.

Nevertheless, several limitations of this study need to be addressed. First, the study is still explorative and correlational; consequently, there is no direct evidence of causality between the variables. The workers involved in our study are not representative of all geographical areas in Italy, which narrows the generalizability. Future research should address this issue by involving workers from different regions of Italy and lines of work. Our results should also be tested in other countries, especially given that the ISC measure is becoming available in multiple languages [24,44]. In this way, cross-cultural invariance of our results could also be tested. In addition, other primary prevention constructs should be investigated together with our model to improve the model itself. Among these are emotional intelligence [45,46], empathy [47,48], positive relational management [49], and workplace relational civility [50]; these areas certainly deserve special attention given the role of advanced relational competencies in enhancing people’s well-being. Moreover, sustainable leadership [51] could plausibly contribute to this framework, as well as adaptively facing the challenges related to sustainability [52]. Finally, the proposed model should be also extended to include variables such as environmental education [53] to give a more comprehensive overview of the phenomenon.

Interestingly, ISC can be increased through specific training [54,55] and for this reason it is particularly suitable for a primary prevention perspective [2,56,57,58]. At this stage, ISC interventions are needed to prove causality between the variables encompassed by our model. Also, interventions based on information and communication technologies (ICT) should be considered, as well as interventions for educators [59], as a viable solution to improve people’s connectedness to nature together with ISC resources. Indeed, ICT interventions based on gamification appear able to enhance people’s awareness of sustainability and sustainable development issues [60]. Nonetheless, their potential could be further expanded by using those social cues, such as reputation, to which people are particularly sensible in virtual environments [61,62]. In strength-based preventative perspectives [63] to improve employees’ well-being, organizations could invest in specific training for enhancing the resources of workers to create ISC, as well as in relation to sustainability and sustainable development. A focus on the promotion of workers’ strengths could foster individual and organizational well-being for more sustainable work environments.

## 5. Conclusions

Overall, this work suggests that ISC could encourage sustainable development within organizations [5,64] by fostering connectedness to nature in terms of well-being in the workplace. In this new digital era, organizations and educators are being called upon to contribute to the achievement of the United Nation’s sustainable development goals [8]. Enhancing those psychological resources [3], as with ISC, in a way that is connected with sustainable development and people’s well-being, is a promising opportunity.

## Figures and Tables

**Table 1:** Descriptive Statistics.

Variable	Mean (s.d.)
Connectedness to nature scale (CNS)	50.27 (10.98)
Work and meaning inventory (WAMI)	50.63 (12.97)
Intrapreneurial self-capital scale (ISCS)	103.05 (11.60)

Note. s.d. = standard deviation.

**Table 2:** Full correlation matrix between connectedness to nature (CNS), the work and meaning inventory (WAMI), and the intrapreneurial self-capital scale (ISCS).

Variable	CNS	WAMI	ISC
**CNS**	1	0.28 **	0.37 **
**WAMI**	0.28 **	1	0.35 **
**ISCS**	0.37 **	0.35 **	1

**: *p* < 0.01.

**Table 3:** Gender differences among selected variables for mediation analysis.

Variable	Women		Men		Gender Differences Cohen’s d
	*M*	*s.d.*	*M*	*s.d.*	
**CNS**	50.05	9.24	50.67	8.52	0.07
**WAMI**	51.27	14.19	49.44	10.39	0.14
**ISCS**	102.00	10.89	104.97	12.64	0.25

Note. M = mean; s.d. = standard deviation.

**Table 4:** Model 1. Mediation analysis statistics.

	F	df	*p*	R^2^
X predicts M	31.05	1, 201	0.001	0.14
X and M predict Y	17.69	2, 200	0.001	0.15
X predicts Y	16.71	1, 201	0.001	0.08
	Student t	df	*p*	β
Path a	5.57	201	0.001	0.47
Path b	4.16	200	0.001	0.32
Path c’	2.43	200	0.016	0.24
Path c	4.09	201	0.001	0.40

df: degrees of freedom.

**Table 5:** Model effect indices.

Total Effect	Direct Effect	Indirect Effect	Partial Standardized Indirect Effect	Total Standardized Indirect Effect
0.40	0.25	0.15	0.01	0.11
